# Magnitude and associated factors of husband involvement on antenatal care follow up in Debre Berhan town, Ethiopia 2016: a cross sectional study

**DOI:** 10.1186/s12884-020-03264-5

**Published:** 2020-09-25

**Authors:** Sisay Shine, Behailu Derseh, Bethlehem Alemayehu, Gebrehiwot Hailu, Hussein Endris, Saba Desta, Yibeltal Birhane

**Affiliations:** 1 Public Health Department, Institute of Medicine and Health Science, Debre Berhan University, Debre Berhan, Ethiopia; 2 Midwifery Department, Institute of Medicine and Health Science, Debre Berhan University, Debre Berhan, Ethiopia

**Keywords:** Husband involvement, Antenatal care, Debre Berhan, Ethiopia

## Abstract

**Background:**

Involving the husband in antenatal care follow up have a crucial role in pregnancy outcome and highly recommended by the world health organization. Data on husbands’ involvement during ANC follow up in Debre Berhan town was scarce. Therefore, the objective of this study was to assess the magnitude and factors associated with the husband involvement in accompanying their wife to ANC follow up.

**Methods:**

A cross-sectional study was conducted during the study period among 405 married men whose wife was pregnant in the last year. A multi-stage sampling technique was used to select the study participants. Data were collected using a pre-tested and structured questionnaire. Odds ratio with 95% confidence intervals were used to assess levels of significance.

**Results:**

More than half the 62.5% (252/405) of the husbands were involved in accompanying their wife in ANC follow up. A majority, 92.3% (374/405) of husbands had good communication with their wife during pregnancy and 88.6% (359/405) of husbands discussed with doctor about the health-related condition of their wife. Age category of husbands 30–39 years old (AOR: 1.9; 95%CI: 1.1, 3.2) and the educational status of husbands being illiterate and primary school (AOR: 1.8; 95%CI: 1.1, 3.1) and secondary school (AOR: 3.1; 95%CI: 1.7, 5.7) were significant predictors on accompanying their wife in ANC follow up.

**Conclusion:**

More than half of the husbands were involved in accompanying their wife to ANC follow up. The age and educational status of the husband had significantly associated with an accompanying their wife to ANC follow-up. Educating husbands on the importance of their involvement during pregnancy increase their participation in ANC follow up.

## Background

The maternal mortality ratio in Ethiopian is high with an estimated 412 deaths per 100,000 live births [1]. Antenatal care (ANC) follow up by skilled health care provider is important to monitor pregnancy and reduce morbidity and mortality risk for the mother and child during pregnancy. In Ethiopia 65% of pregnant women attend ANC at least once and only 32% attend the recommended four and more visits [1, 2].

Different studies conducted in developing country indicated that husband involvement in antenatal care follow up can improve maternal pregnancy outcomes [3, 4] and highly recommended by the World Health Organization (WHO) [5]. It was recognized in 1994 on Population Development International Conference in Cairo [6]. In many developing countries men are the key decision-makers and chief providers of economic resources for their wives. Their role is highly influential in women’s choice for health care seeking behavior [5, 7]. Support from husbands offered to their wife have many benefits including increased self-esteem, better health, reduction in the duration of labour, anxiety and operative deliveries, early bonding with their newborns and improvement in maternal nutrition [8]. Studies conducted in developing country including Ethiopia indicated that husband involvement on accompanying their wife in ANC follow up was at infancy stage [9]. This low involvement is associated with factors such as lack of awareness about the importance of their participation [9], educational status of husband [10, 11], household monthly income [12] and parity [9, 13].

Despite the fact that men involvements during ANC follow up have a big role on pregnancy outcome, magnitude of involvement of men was low in Ethiopia. No previous study was attempted to assess the magnitude and determinants of husbands’ participation in ANC follow up in Debre Berhan Town. Therefore, the aim of this study was to measure the magnitude and factors associated husband’s involvement in their wives ANC attendance. The findings from the study will help recommend some interventions to engage men in maternal and child health services.

## Methods

### Study area

This study was done in Debre Berhan town which is located 130 km away from Addis Ababa. Based on the Debre Berhan town health administration office report, the current population of the town is 103,450 of whom 46, 553 men, and 56,897 women. In the town, 20.23% of the total population estimated to be reproductive age group women and estimated pregnant women are around 77,483 in 2016. Within the town, there is one public and one private hospital, three public health centers, fifteen private clinics, one university, two colleges, and fifteen industries [14].

### Study design and period

A cross-sectional study design was conducted in Debre Berhan town from March 1–30, 2016.

### Study population

The source population for this study was married men who live in Debre Berhan town and married men who live in the selected kebeles of Debre Berhan town were the study population. Married men whose partner were pregnant in the last 1 year period and volunteer to participate were inclusion criteria.

### Sample size determination

The sample size was determined based on a single population proportion formula by taking an assumption of 60.4% of husbands were involved during pregnancy from Mekelle town [15] community-based study, 5% margin of error, and 95% confidence level.

$$ n=\frac{{\left({Z}_{\raisebox{1ex}{$\alpha $}\!\left/ \!\raisebox{-1ex}{$2$}\right.}\right)}^2P\left(1-P\right)}{d^2} $$

$$ n=\frac{(1.96)^2\ast 0.604(0.396)}{0.05^2}=368 $$

After adjusting for non-response rate of 10% the final required sample size was 405.

### Sampling procedure

**Fig. 1:**
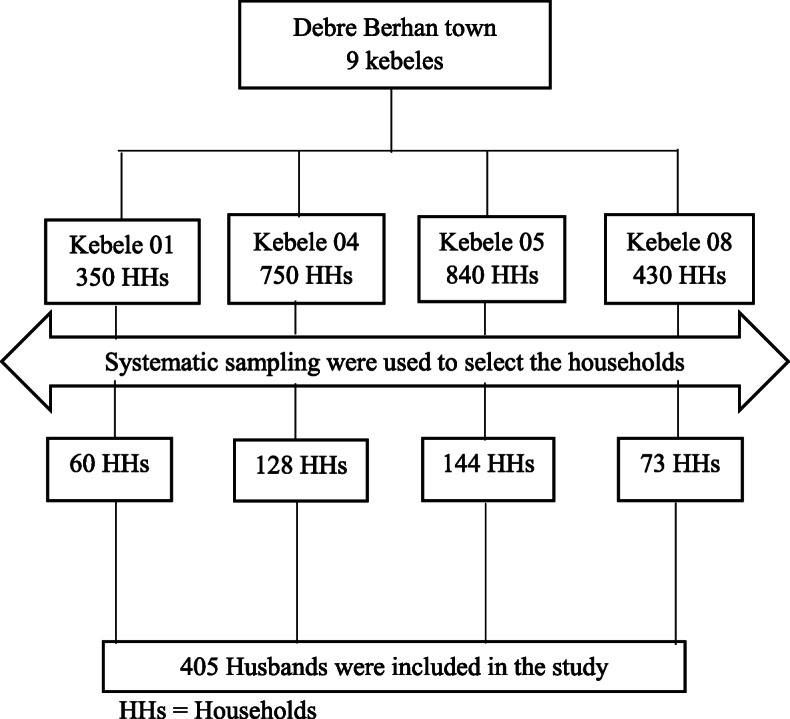
Schematic representation of sampling procedure on assessment of magnitude and associated factors of husband involvement on antenatal care follow up in Debre Berhan Town, Ethiopia 2016

Respondents were identified and approached through a multi-stage sampling technique. At the start, four kebeles were identified from the Debre Berhan town through a lottery method. Then, an assessment was done to identify eligible households. In the end, households were recruited through a systematic sampling technique. The husband was selected from each selected households for the interview (Fig. 1).

### Data collection tool and methods

Data were collected by seven trained BSc midwives and three supervisors deployed for supervision. A structured questionnaire was adopted in English after reviewing different kinds of literature and guidelines [7, 9, 16, 17]. English version questionnaire was translated to Amharic language and again translated back to English by experts. Before the actual data collection, pre-testing was performed and necessary modifications were made on the questionnaire. Finally, Data were collected by face to face interview.

### Outcome variable

In this study, level of husband involvement on accompanying their wife in ANC follow up was the outcome variable.

The husband’s involvement in escorting their wife in ANC follow up was defined as the presence of the husband in any one of the antenatal care visits with his wife. The data collector asks the study participants “Did you escort your wife during her ANC visits?” With options yes/no to determine if the husband had involved in any antenatal care visits with his wife. For the response, we used Yes = 1, and No = 2 for assessment. We didn’t have missing values for the response.

### Independent variables

The independent variables in this study were chosen based on existing literature on husband’s involvement on accompanying their wife in ANC follow up. Age of husbands was recorded as a continuous variable and then recoded into categories in the assessment data set. Educational status of husbands was recorded as categorical variable: (i) can’t read and write, (ii) primary school (1–8 grade), (iii) secondary school (9–12 grade), (iv) certificate and above. A type of marriage also recorded as categorical variable (i), religious marriage, (ii) civil marriage, (iii) traditional marriage. Household monthly income in this study was recorded as a continuous variable and recorded as below the mean and above and equals to the mean. Availability of TV and Radio at household level was recorded as categorical variable (i) yes, (ii) no. Husband thinks pregnancy is the women’s duty recorded as husband thinks so (i) yes and if husband doesn’t think like that (ii) no. Husband believes child birth is a natural phenomena if husband think so (i) yes and if husband doesn’t think like that (ii) no. Distance from home to a health facility in this study was recorded as a continuous variable and recorded as below and equal to the mean and above the mean. Health worker cooperation on husband’s involvement was recorded by asking husband, whether health workers are (i) cooperative, (ii) not cooperative. Problem during pregnancy recorded as any problem that the wife faced during pregnancy, it may be pregnancy complications or any non-clinical issues (i) yes, (ii) no. Know health facility that provides ANC service recorded as by asking husband know health facility that provide ANC service (i) yes, (ii) no.

### Statistical analysis

Data were cleaned and entered into the Epi-info computer software package version 3.5.1. and then exported to SPSS Window Version-16 for analysis. To identify the factors associated with husband involvement in ANC follow up binary and multiple logistic regression analysis were performed. Variable with *P*-value < 0.05 in the binary logistic regression analysis were included in the multiple logistic regression analysis. To select variables in multiple logistic regression analysis enter method was used. To assess the existence of correlation among predictor variables multicollinearity test was performed Hosmer and Lemeshow goodness of fit to the final model was also checked and was found fit. The strength of the association between the predictor variable and outcome variables was assessed by using adjusted odds ratios along with 95% confidence intervals. A *P*-value of less than 0.05 was declared as statistically significant. Finally, the data were presented using statements, tables, and figures.

## Results

### Socio-demographic characteristics of the study participants

A total of 405 study participants, were included in the study with a response rate of 100%. The mean age of respondents was 33.63 (SD + 6.11) years. About 67.7% (274/405) respondents were orthodox and the majorities 77.3% (313/405) were an Amhara ethnic group. More than half 51.1% (207/405) of respondents, educational status was a certificate and above and 56.5% (229/405) were government employees (Table 1).

**Table 1:** Socio-demographic characteristics of husband in Debre Berhan town, Ethiopia 2016

Variable	Frequency	Percent
Age		
20–29	117	28.9
30–39	218	53.8
> 40	70	17.3
Religion		
Orthodox	274	67.7
Muslim	69	17.0
Protestant	62	15.3
Ethnicity		
Amhara	313	77.3
Oromo	70	17.0
Other	22	5.7
Educational status		
Illtrate & primary school (1–8 grade)	128	31.6
Secondary school (9–12 grade)	70	17.3
Certificate and above	207	51.1
Occupation		
Government worker	229	56.5
Merchant	109	27.0
Daily laborer	67	16.5
Types of marriage		
Religious marriage	105	25.9
Civil marriage	190	46.9
Traditional marriage	110	27.2
Have TV at household		
Yes	373	92.1
No	32	7.9
Have Radio at household		
Yes	340	84.0
No	65	16.0
Household monthly income (in Ethiopian Birr)		
< 4600	223	55.1
> 4600	182	44.9

### Attitude and health related characteristics of the respondents

More than half, 56.1% (225/405) of the husbands believe that childbirth was the natural phenomena for women. The majority of 69.2% (280/405) husbands think that health workers were cooperative on the involvement of husbands on accompanying their wife in ANC follow up. About 64.6% (262/405) of husbands’ partner had two and more pregnancy experiences and 51.4% (208/405) had two and more than two total live births (Table 2).

**Table 2:** Attitude and health related characteristics of respondents in Debre Berhan town, Ethiopia 2016

Variable	Frequency	Percentage
Husband think pregnancy is women’s duty		
Yes	140	34.6
No	265	65.4
Husband believe child birth is natural phenomena		
Yes	227	56.1
No	178	43.9
Health workers cooperation on husband involvement		
Cooperative	280	69.2
Not cooperative	125	30.8
Distance from home to health facility (in meter)		
< 600	251	61.9
> 600	154	38.1
No. of total pregnancy by husbands’ wife		
< 2	262	64.6
> 2	143	35.4
No. of total live birth by husbands’ wife		
< 2	208	51.4
> 2	197	48.6
Problem face during pregnancy		
Yes	96	23.7
No	309	76.3
Husband know health facility that provide ANC service		
Yes	326	80.5
No	79	19.5

*ANC* Antenatal Care

### Husband involvement during pregnancy

The entire study participants’ wife visited the ANC clinic at least once in their last pregnancy and only 11.4% (46/405) wives had the recommended four and more visits. This study found that the magnitude of the husbands’ involvement in accompanying their wife in ANC follow up was 62.2% (252/405), of these more than half 50.4% (127/252) were present with their wife only once. The majority of study participants, 92.3% (374/405) had good communication with their wife during pregnancy. About 88.6% (359/405) of husbands discussed with the doctor on the health-related condition of their partners. More than half of the study participants, 62.9% (255/405) involved in the decision making where their wife should attend ANC follow-ups during pregnancy. About 88.6% (359/405) of husbands involved in purchasing household daily consumption goods (Table 3).

**Table 3:** Husband involvement during pregnancy in Debre Berhan town, Ethiopia 2016

Variable	Frequency	Percentage
Number of ANC visit by wives		
Once	158	39.0
Twice	105	25.9
Three times	96	23.7
Four and more	46	11.4
Accompanied their wives in ANC follow up		
No	153	37.8
Yes	252	62.2
Number of ANC visit with husband (*n* = 252)		
Once	127	50.4
Twice	62	24.6
Three times	58	23.0
Four and more	5	2.0
Had good communication with wife		
Yes	374	92.3
No	31	7.7
Discussed with doctor about their wives health		
Yes	359	88.6
No	46	11.4
Decision making where to attend ANC follow up		
Yes	255	63.0
No	150	37.0
Involved on household daily consumption goods purchase		
Yes	359	88.6
No	46	11.4

*ANC* Antenatal Care

### Factors associated to husband unaccompanied their wives in ANC follow up

In the binary logistic regression analysis, age of the husband, educational status of the husband, household monthly income, number of pregnancy, the number of live birth children, and husband know where to provide ANC service was significantly associated with husband unaccompanied their wife in ANC follow up. The result from multiple logistic regression analysis revealed that age and educational status of husbands were significantly associated with unaccompanied their wife in ANC follow up. The odds of unaccompanied their wife in ANC follow up among husbands in the age category of 30–39 years were 1.9 times higher (AOR: 1.9; 95%CI: 1.1, 3.2) compared from the age category of 20–29 years. Moreover, the odds of unaccompanied their wife in ANC follow up among husbands educational status of illiterate and primary school and secondary school were 1.8 times (AOR: 1.8; 95%CI: 1.1, 3.1) and 3.1 times (AOR: 3.1; 95%CI: 1.7, 5.7) higher compared to those who had a certificate and above, respectively (Table 4).

**Table 4:** Binary and multiple logistic regression analysis on determinants of husbands unaccompanied their wife in ANC follows up in Debre Berhan town, Ethiopia 2016

Variable	Unaccompanied their wife in ANC follow up		COR (95%CI)	AOR (95%CI)
	Yes	No		
Age of husband				
20–29	28	89	1.00	1.0
30–39	93	125	2.4 (1.4, 3.9)	1.9 (1.1, 3.2)*
> 40	32	38	2.7 (1.4, 5.0)	1.9 (0.9, 3.9)
Educational status				
Illiterate & primary school	56	72	2.0 (1.3, 3.2)	1.8 (1.1, 3.1)*
Secondary school (9–12 grade)	39	31	3.2 (1.8, 5.7)	3.1 (1.7, 5.7)**
Certificate and above	58	149	1.0	1.0
Household monthly income (in Ethiopian Birr)				
< 4600	93	130	1.5 (1.7, 2.2)	1.0 (0.6, 1.6)
> 4600	60	122	1.0	1.0
Husband know health facility that provide ANC service				
Yes	128	194	1.0	1.0
No	25	58	1.5 (1.1, 2.5)	0.7 (0.4, 1.2)

* Significant at *P* < 0.05, ** Significant at *P* < 0.001, *ANC* Antenatal Care, *COR* Crude Odd Ratio, *AOR* Adjusted Odd Ratio

## Discussion

The Ethiopian government has built into an impressive work through health extension workers to improve utilization of ANC service among pregnant women by increasing husband involvement [18]. But, this effort of the government needs to evaluate though research. Therefore, this research also one indicator of the status of the husband’s involvement in pregnancy service utilization. This study identified that the magnitude of husband involvement in escorting their wife in ANC clinics was 62.2%. This was congruent with the previous study reported in Myanmar (64.8%) [13] and in Ethiopia (50.8%) [19]. However, the finding of this study was higher than the study conducted in Ghana (35%) [16], Nepal (39.3%) [7], Uganda (6.0%) [20], and in Ethiopia (19.7%) [9]. In this study, the variation might be due to the fact that most husbands had good communication with their wife (92.3%), health professionals (88.6%) and had an exposure to mass media like radio (84%) and television (92.1%) which lead them to have a good understanding of the importance of involvement during ANC follow-ups. This study was conducted near (130 km) the capital city of Ethiopia and rapidly growing town these have great contribution on the increment of husband involvement on ANC follow up because living in an urban or near to it had access to maternal and child health services.

Husbands in the age category of 30–39 years old increased the odds of unaccompanied their wife in ANC follow up compared from age categories of 20–29 years old. The prevalence of husband unaccompanied their wife to ANC visit in this study was higher among older participants. It was inconsistent with the study conducted in Nepal [21] and Ethiopia [22]. The probable reasons for the difference could be since today Ethiopian education policy focused on the younger population most older participants were not educated and they may also have less access to social media (like Facebook, Twitter, Instagram, etc.), which can easily access the importance of accompanying their wife to ANC follow up than younger participants. Moreover, this youth-friendly service, which was newly implemented in Ethiopian health service the system, may also have a big role in maternal and child health service development [23].

This study showed that low educational status of the husband being illiterate and primary and secondary school increased the odds of husband unaccompanied their wife in ANC follow up compared from a husband who have a certificate and above. It was consistent with the study conducted in Nepal, Ghana, India and Ethiopia [16, 21, 24, 25]. The possible reasons might be that low educated husbands have low potential to engage in health promotion message or might not have better insight into the benefits of presenting with their wife in ANC follow up. Moreover, low educated men are less likely to discuss with their wife about the maternal health issues than their counterparts [23].

In this study, we had limitations that we should be noted. The use of a cross-sectional study may not create a true causal relationship between husband involvement in accompanying their wife to ANC clinics and their risk factors. Qualitative data were not included to explore some associated factors and to triangulate the findings of the quantitative data with qualitative data.

## Conclusion

In this study, more than half of the husbands were involved in accompanying their wife to antenatal care follow up. Since the study was conducted in an urban area, it was relatively high but, encouraging more support by husbands during pregnancy and childbirth can lead to positive birth outcomes for the mother and baby and improved maternal and child health. Specifically, improving access to formal education could help to know the benefits of accompanying their partner to antenatal care follow up. Lastly, we recommend conducting further longitudinal and qualitative studies on husbands’ participation in maternal care services.

## Supplementary information

**Additional file 1.** Questionnaire on Husband involvement in ANC follow up study

## Data Availability

The datasets used and/or analyzed during the current study available from the corresponding author on reasonable request.

## Abbreviations

ANC: Antenatal care
AOR: Adjusted odd ratio
SD: Standard deviation
SPSS: Statistical package for social science
WHO: World health organization
