# Supplementary material for: Magnitude and associated factors of husband involvement on antenatal care follow up in Debre Berhan town, Ethiopia 2016: a cross sectional study
Source: BMC Pregnancy Childbirth. 2020 Sep 25;20:567. doi: 10.1186/s12884-020-03264-5 (PMC7519520; doi:10.1186/s12884-020-03264-5)
Supplement: Supplementary file 1 — Additional file 1. Questionnaire on Husband involvement in ANC follow up study [file 12884_2020_3264_MOESM1_ESM.docx]

**Questionnaire on Husband involvement in ANC follow up study**

| **NO** | **QUESTIONS** | **RESPONSE** | **Remark** |
| --- | --- | --- | --- |
| **Socio-demographic characteristics of the study participants** | | | |
| 101 | How old are you? | ________ (in full years) |  |
| 102 | What is your religion? | 1. Orthodox 2. Muslim 3. Protestant |  |
| 103 | What is your ethnicity? | 1. Amhara 2. Oromo 3. Other |  |
| 104 | What is your educational status? | 1. Can’t read and write 2. Primary school (1-8 grade) 3. Secondary school (9-12 grade) 4. Certificate and above |  |
| 105 | What is your Occupation? | 1. Government worker 2. Merchant 3. Daily laborer |  |
| 106 | What type of marriage did you have? | 1. Religious marriage 2. Civil marriage 3. Traditional marriage |  |
| 107 | Do you have TV in your home? | 1. Yes 2. No |  |
| 108 | Do you have radio in your home? | 1. Yes 2. No |  |
| 109 | Please can tell your home average monthly income? | ___________ (in Ethiopian birr) |  |
| **Attitude and health related characteristics of husband** | | | |
| 201 | Do you think pregnancy is women’s duty? | 1. Yes 2. No |  |
| 202 | Do you believe child birth is natural phenomena? | 1. Yes 2. No |  |
| 203 | Do you think that health workers are cooperation on husband involvement | 1. Cooperative 2. Not cooperative |  |
| 204 | Can you tell us distance from home to health facility? | ______________ (in meters) |  |
| 205 | Can you tell us number of total pregnancy by your wife? | __________ (No. of pregnancy) |  |
| 206 | Can you tell us number of total live birth children by your wife? | __________ (No. of children) |  |
| 207 | Is there any problem face on your wives during pregnancy? (*pregnancy complications or any non-clinical problems*) | 1. Yes 2. No |  |
| 208 | Do you know health facility that provide ANC service? | 1. Yes 2. No |  |
| **Husband involvement during pregnancy** | | | |
| 301 | Did your wife have ANC follow up? | 1. Yes 2. No |  |
| 302 | How many number of ANC visits had your wife during pregnancy? | __________ (No. of ANC visits) |  |
| 303 | Did you accompany your wife during her ANC visits? | 1. Yes 2. No | If Q303 is no skip to Q305 |
| 304 | How many number of ANC visits you had with your wife? | ________(No. of ANC visits with husband) |  |
| 305 | Do you have good communication with your wife? | 1. Yes 2. No |  |
| 306 | Do you have any discussion with doctor about your wife health? | 1. Yes 2. No |  |
| 307 | Did you participate on decision making activity where to attend ANC follow up for your wife? | 1. Yes 2. No |  |
| 308 | Did you involved on household daily consumption goods purchase? | 1. Yes 2. No |  |
